# Surgery at the frontline at the time of the COVID‐19 outbreak

**DOI:** 10.1111/1759-7714.13666

**Published:** 2020-09-16

**Authors:** Alberto Testori, Ugo Cioffi, Michele M. Ciulla, Edoardo Bottoni, Umberto Cariboni, Gianluca Perroni, Marco Alloisio

**Affiliations:** ^1^ Department of General and Thoracic Surgery Humanitas Research Hospital Rozzano Italy; ^2^ Department of Surgery University of Milan Milan Italy; ^3^ Laboratory of Clinical Informatics and Cardiovascular Imaging, Department of Clinical Sciences and Community Health University of Milan Milan Italy

## Abstract

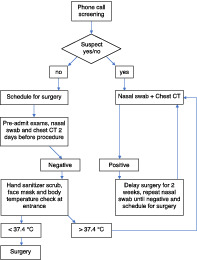

## Introduction

The SARS‐CoV‐2, previously named 2019 novel coronavirus (2019‐nCoV), is an enveloped positive‐sense single‐stranded RNA virus causing bilateral pneumonia with respiratory distress syndrome and was first described in late December 2019 in Wuhan city, China.^1^ Since coronavirus disease 19 (COVID‐19) has developed into a pandemic, some forms of contagion mitigation, borrowed from China, have been gradually adopted in other countries and, first, in Italy.^2^ However, despite recommendation from the World Health Organization (WHO), no agreement has been reached on appropriate preventive actions and therefore differences do exist from nation to nation.^3^ Most health systems appear to be hit hard by two problems: the availability of hospital beds, especially those with intensive care units (ICUs), and an increasing number of infections between health personnel thus affecting the workforce and morale of staff members. Thus, as previously described in China, a reduction or even a suspension of elective surgery in all affected countries is expected.^4^, ^5^ To counteract this phenomenon, our hospital along with surgical teams created new pathways and guidelines both for patients and healthcare professionals to offer the best level of care in order to limit the detrimental effect on surgical volume caused by the pandemic. We hereby describe in detail our results.

### Report of the activity at the outbreak

**Figure 1:**
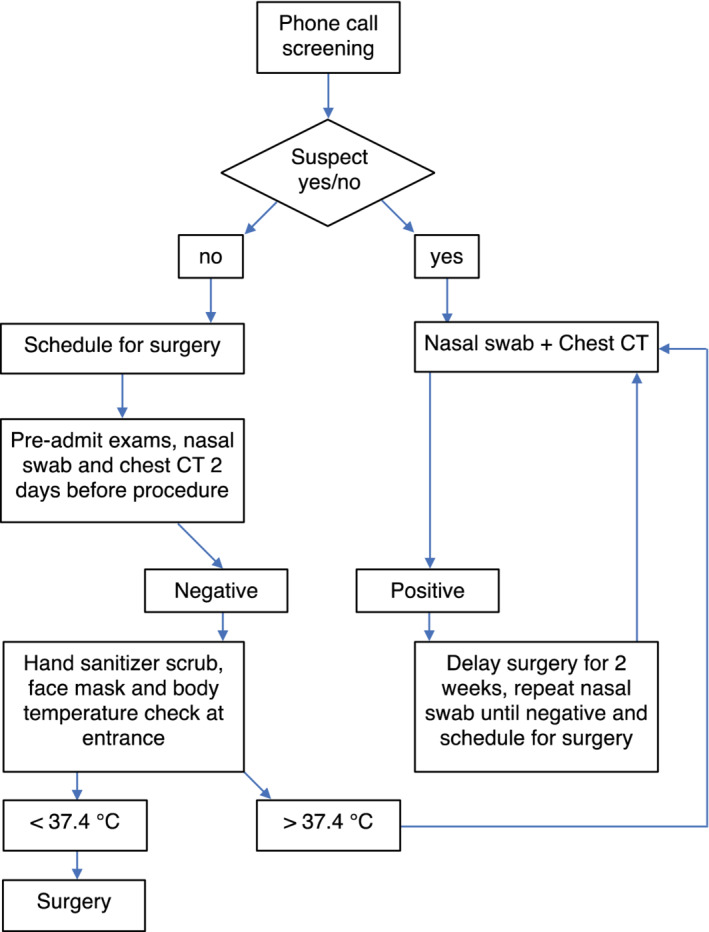
Showing flowchart created by our hospital.

From 17 to 23 February 2020, we performed 10 breast and 21 thoracic surgeries with a total of 31 surgeries. However, due to the enormous increase in COVID‐19 patients requiring hospitalization, we were forced to reduce the number of daily procedures reduced to a minimum between February 24 to March 1, with only two and nine cases of breast and lung cancer, respectively. Meanwhile, our hospital underwent a substantial change, creating a brand new flowchart (Fig 1). Doctors were subsequently asked to screen any suspicious symptoms by calling patients before hospitalization. A body temperature check along with facial mask and hand sanitizer scrub were mandatory for everyone at the hospital entrance and relatives were not allowed to accompany or visit patients. Moreover, the building was divided into two main areas with dedicated pathways: a “clean” one for oncological patients requiring either surgery or chemoradiotherapy and a “COVID‐19” area for those who had been assessed or with a suspected viral infection. The main purpose was to create distinct routes than did not cross, thereby limiting in‐hospital spread. A nasal swab was performed on patients scheduled for surgery together with a computed tomography (CT) chest scan to exclude any asymptomatic carriers two days before surgery. Our Breast Surgery Unit (BSU) optimized procedures when possible, preferring a day surgery regimen to reduce the infection risk to a minimum. Patients were accepted and subsequently discharged directly from the surgical unit, avoiding ward admission. If an overnight stay was necessary, a single room was offered. Regarding lung surgery, urgent oncological procedures (eg, resectable T3/T4 tumors or N1/N2 disease with or without induction chemotherapy) were prioritized over those that were nonurgent (eg, T1a N0 or pure ground‐glass opacities [GGO]). If there was a case of a critical patient from the emergency room who had not received a nasal swab test, a dedicated operating room with special suits was available. Coordination and communication between doctors and medical administration office was the stronghold during the pandemic. Daily updates on nationwide cases, workflow modification and guidelines were provided by email or through the institutional website.

## Results

From 2 March to 27 May 2020, we performed 113 and 143 procedures for breast and lung cancer, respectively. Regarding breast surgery, 17 out of 113 (15.04%) were mastectomy, of which 15 were reconstructive surgery and two were other procedures. The remaining 96 (84.96%) were quadrantectomy patients with sentinel node biopsy in 88 cases. When considering lung surgery, 129 out of 143 (90.21%) were major procedures with 90 lobectomies (69.77%), 25 wedge resection (19.38%), 11 anatomical segmentectomy (8.53%) and three pleurectomies for mesothelioma (2.32%). The remaining 14 (9.79%) were diagnostic procedures on the pleura, mediastinum and chest wall. Two patients in the breast group were positive for SARS‐CoV‐2 with no symptoms and subsequently fully recovered. One patient in the lung cancer group died due to COVID‐19 after their surgical procedure. Among members of the surgical unit, six out of 41 were infected with COVID‐19. One of them needed hospitalization and was discharged after seven days with no need for ICU, two had only slight symptoms and three were asymptomatic.

## Discussion

Here, we show that, despite the COVID‐19 pandemic infection, our successful model and precautions allowed us to continue treating cancer patients safely with a reproductible method in other similar surgical centers. Furthermore, dedicated hospitals for treatment of COVID‐19 can guarantee appropriate treatment for oncological patients thus separating those two diseases.^6^ The fear of the general population of infection has led to a reduction in hospital usage, and therefore an upstaging is expected due to an increase in late diagnoses.
